# Platelet indices and the risk of pulmonary arterial hypertension: a two-sample and multivariable Mendelian randomization study

**DOI:** 10.3389/fcvm.2024.1395245

**Published:** 2024-08-08

**Authors:** Yinuo Li, Xi Liu, Qian Hong, Rui Xu

**Affiliations:** ^1^ First School of Clinical Medicine, Shandong University of Traditional Chinese Medicine, Jinan, China; ^2^ Department of Cardiology, Central Hospital Affiliated to Shandong First Medical University, Jinan, China

**Keywords:** pulmonary arterial hypertension, platelet count, plateletcrit, mean platelet volume, platelet distribution width, Mendelian randomization, causal relationship

## Abstract

**Background:**

Recent epidemiological studies have indicated a correlation between platelet indices and pulmonary arterial hypertension (PAH), yet the causality between them remains unclear. To explore the causal relationship between four platelet indices and PAH, with the aim of providing a theoretical basis for clinical prevention and treatment.

**Methods:**

Single-nucleotide polymorphisms (SNPs) associated with platelet-related traits were selected as exposure factors from published genome-wide association studies (GWAS), including: platelet count (PLT), plateletcrit (PCT), mean platelet volume (MPV), and platelet distribution width (PDW). Summary-level data for PAH were obtained from the FinnGen study (248 cases and 289,117 controls). Two-sample and multivariable Mendelian randomization (MR) analyses were conducted to assess the causal relationship between exposure factors and the risk of outcomes. The inverse variance weighted (IVW) method was utilized as the primary MR analysis approach, supplemented by weighted median, mode-based estimation, MR-Egger regression, and the MR Pleiotropy Residual Sum and Outlier (MR-PRESSO) test to detect and adjust for pleiotropy, ensuring the reliability of the results through sensitivity analysis.

**Results:**

(1) The IVW results from the two-sample MR analysis showed a positive causal association between PLT and the risk of developing PAH [(OR = 1.649, 95%CI: 1.206–2.256, *P* = 0.0017)], with the sensitivity analysis confirming the robustness of the causal relationship. The MR-Egger intercept analysis did not detect potential pleiotropy (*P* = 0.879). (2) The MVMR results showed no statistically significant causal relationship between these four markers and the risk of developing PAH. After adjusting for collinearity, a direct positive causal association was observed between PLT and the risk of developing PAH (OR = 1.525, 95%CI: 1.063–2.189, *P* = 0.022).

**Conclusion:**

The positive correlation between PLT and the risk of PAH suggests that correcting elevated platelet levels may reduce the risk of developing PAH.

## 1 Introduction

Pulmonary arterial hypertension (PAH) is a progressive disease characterized by increased pulmonary arterial pressure and pulmonary vascular resistance, with a prevalence of approximately 10.6 cases per 1 million adults (1). PAH is characterized by persistent high pulmonary arterial pressure and increased pulmonary vascular resistance, which, if left untreated, may eventually lead to right heart failure and patient death. With advancements in basic research, numerous clinical randomized controlled trials, the establishment of guidelines, and the implementation of new treatment protocols, significant improvements have been achieved in the treatment of patients with pulmonary hypertension; however, the mortality rate remains high. Challenges persist, including an unclear pathogenesis, lack of early diagnostic and prognostic assessment methods, and the need for optimized treatment and management strategies.

Platelets play a key role in the pathogenesis of PAH, influencing a variety of mechanisms associated with PAH pathogenesis, including thrombosis, vasoconstriction, and remodelling, all of which are core processes in PAH pulmonary vascular pathology (2). Previous studies have shown that several platelet indices, including platelet count (PLT), plateletcrit (PCT), mean platelet volume (MPV), and platelet distribution width (PDW), are strongly associated with the onset, progression, and prognosis of PAH (3–5). Although numerous studies have identified an association between platelet indices and PAH, the findings have been inconsistent, and the causal relationship between changes in platelet indices and the development of pulmonary hypertension has been unclear owing to limitations in traditional research methodologies.

Mendelian randomization (MR) analysis, as a method similar to natural randomized clinical trials, is capable of robustly assessing the causal relationship between exposure and outcome by using genetic variants such as single nucleotide polymorphisms as instrumental variables (IVs) (6). The advantage of MR analysis is that, due to the random assignment property of alleles, it can effectively reduce the influence of confounding factors and avoid reverse causality bias, thus yielding more reliable causal effects (7, 8). Multivariate Mendelian randomization (MVMR) is an extension of the MR approach, which estimates the independent direct effect of each exposure on a single outcome by analyzing the genetic variants of multiple potentially relevant exposures, thus better controlling for correlations between them and potential confounders (9).

For rare diseases such as PAH, which are difficult to conduct large-scale clinical trials, MR studies have unique advantages, and scholars such as Steeve Provencher have called for more MR studies in the field of PAH (10). Therefore, we collected large-scale data on four typical platelet indices and PAH from the Genome-Wide Association Study (GWAS), explored the correlation between individual platelet indices and PAH using univariate MR analysis, and further estimated the independent effects of each platelet index on PAH by using the MVMR approach. The aim is to assess the potential genetic causal associations and provide a scientific basis for the early diagnosis and prognostic treatment of PAH in the future.

## 2 Materials and method

### 2.1 Research design

We investigated the associations of PAH and platelet traits in men and women of European descent using MR. Univariable MR was performed to explore the causal relationship between PAH and 4 platelet indices, then multivariable MR was used to further assess the direct effect of platelet indices on PAH. IVs selected for the study were mandated to satisfy three criteria: (1) significantly associated with the exposure factor, (2) independent of confounding factors, (3) influencing the outcome variable exclusively through the exposure factor (11).

Given that this research was conducted using publicly available data for statistical analysis, ethical approval was not necessitated.

**Figure 1:**
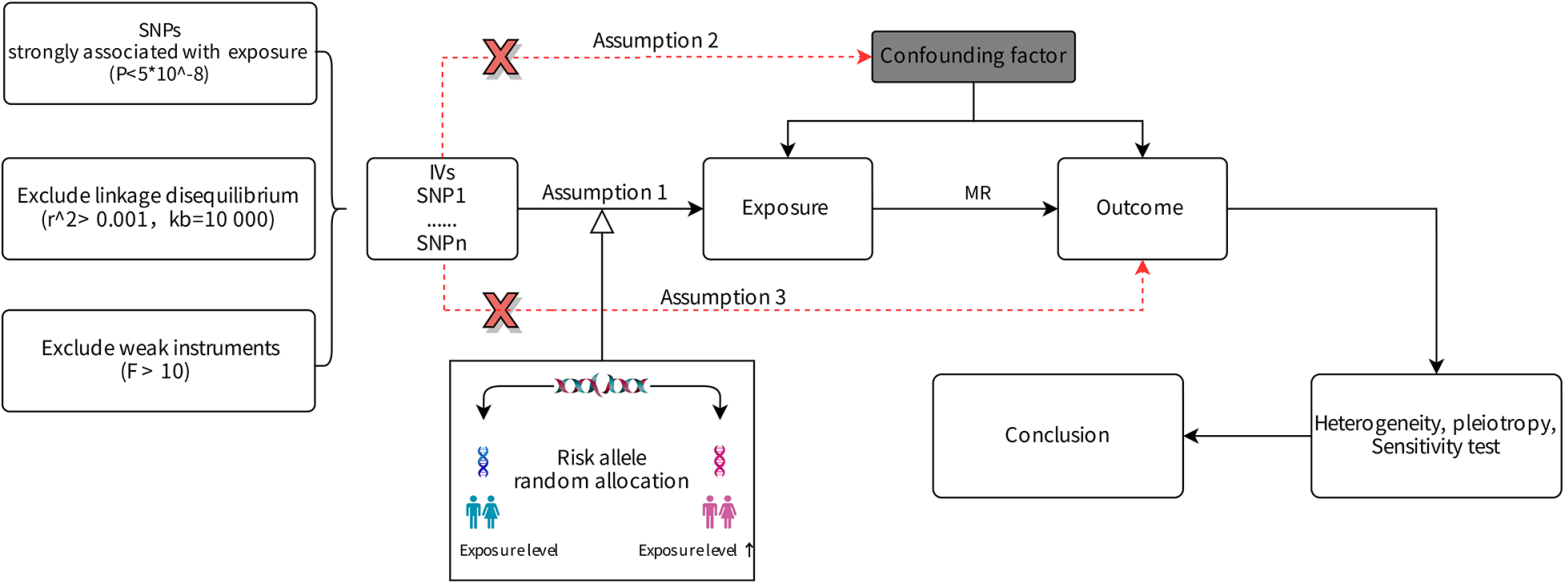
Design of the Mendelian randomization study.

Figure 1 illustrates these criteria and the research design.

### 2.2 Data collection and sources

#### 2.2.1 Data acquisition

Genetic tools for platelet indices were selected from the GWAS by Vuckovic D et al. (12). This study analyzed genome-wide results from 408,112 participants of European ancestry using data from the UK Biobank and explored 29 genetic variants associated with the platelet phenotype, and we chose four indices associated with the platelet phenotype as exposure factors: PLT, PCT, MPV, PDW.

We extracted the genetic associations of IVs with PAH from FinnGen Study. The FinnGen study is a large-scale genomics initiative that has analyzed over 500,000 Finnish biobank samples and correlated genetic variation with health data to understand disease mechanisms and predispositions. The project is a collaboration between research organizations and biobanks within Finland and international industry partners (13). The FinnGen study included 248 individuals with PAH and 289,117 controls.

For details of data sources, refer to Supplementary Table S1.

#### 2.2.2 SNP selection

1. SNPs significantly associated with the exposure factors across the genome (*P* < 5 × 10^−8^) were selected from GWAS data. Criteria of *r*^2^ < 0.001 and a distance threshold of 10,000 Kb were applied to mitigate linkage disequilibrium (LD) biases affecting the study. For multivariable MR analysis, the selection of IVs entailed aggregating and clustering all SNPs with genome-wide significance based on their lowest *P*-value in association with any trait, targeting pairwise LD *r*^2^ < 0.001. This process was facilitated by the TwoSampleMR package. To mitigate weak instrument bias, the F statistic was calculated for each SNP individually, excluding weak instruments with an *F* statistic <10 (14). The F statistic is calculated using the formula: $F\;=\;(N-2)\;\times \;R^{2}/\left(1-R^{2}\right)$, where *N* is the sample size of the exposure GWAS study, and *R*^2^ is the variance in exposure explained by each instrumental variable. *R*^2^ is derived from 2(beta^2^) × eaf × (1-eaf), with EAF denoting the allele frequency of the mutation, and beta representing the effect size of the allele.
2. The pathogenesis of PAH is complex, and early studies have pointed to its association with connective tissue disease, HIV infection, and congenital heart disease, among other factors (1). To ensure the validity of the IVs and to rule out potential confounders, we performed an in-depth query of all selected IVs using the LDlink database (https://ldlink.nih.gov/?tab=ldtrait) to identify whether there were SNPs associated with confounders.
3. Palindromic SNPs were excluded, finalizing the IVs included in this study.

### 2.3 Statistical analysis

#### 2.3.1 MR analysis

After identifying the IVs, this study employs two-sample MR analysis, primarily utilizing the inverse variance weighting (IVW) method for statistical analysis. If Cochran's *Q* test detects no heterogeneity, the IVW fixed-effect model will be employed. In the presence of heterogeneity, the IVW random-effects model will be applied (15, 16).

The IVW method is also one of the most commonly used methods in MR analysis and can provide relatively stable and accurate causal relationships (16). But it may overlook invalid IVs and pleiotropic effects. Therefore, this study incorporates four complementary methods: the Weighted Median Estimator, Weighted Mode, MR-Egger regression model, and Simple Mode (17, 18). These methods aim to refine the estimates derived from the IVW method.

Given the genetic and phenotypic correlations among platelet indices, the multivariable IVW method was employed to disentangle and compare the effects of related platelet indices on PAH. Subsequently, collinearity checks were performed and the MVMR results were readjusted using the lasso method. Effect estimates are reported as odds ratios (OR) and 95% confidence intervals (CIs). In univariate MR analyses, causal effect results are deemed to have strong evidence of a causal relationship at *P* < 0.0125 (after Bonferroni correction) in light of multiple testing concerns. Associations with a *P*-value below 0.05 but above 0.0125 are considered to provide suggestive evidence. Due to the adjusting nature of multivariable MR analysis, sensitivity tests, and *P*-value adjustments do not apply to this analysis method (19).

All statistical analyses were conducted using R statistical software, including the TwoSampleMR and MR-PRESSO packages (R version 4.3.2, R Foundation for Statistical Computing, Vienna, Austria, 2022; https://www.R-project.org).

#### 2.3.2 Sensitivity analysis

The MR-Egger regression method and the pleiotropy residual sum and outlier (PRESSO) test are used to identify and adjust for pleiotropy (20). MR-Egger tests and accounts for unbalanced pleiotropy by estimating the combined causal effects from multiple individual variants using summary data (18).

Cochran's Q statistic is employed to assess heterogeneity among the estimates for each SNP. Cochran's Q statistic provides evidence of heterogeneity caused by pleiotropy or other reasons (21). The Q_*p* value > 0.05 indicates the absence of heterogeneity.

Sensitivity analysis, employing the leave-one-out approach, evaluates the impact of individual SNPs on causal association estimates (22). Funnel plots are utilized to detect result bias, facilitating the examination of potential impacts from outlier and pleiotropic SNPs on causal estimates.

## 3 Results

### 3.1 Selection of IVs

In the present study, IVs for four platelet indices were screened according to established screening criteria. In univariate MR of PLT to PAH, 538 significantly associated SNPs (*P* < 5 × 10^−8^) were screened, three incompatible alleles (rs111909349, rs150095649, rs62251184) were further excluded after merging with the PAH dataset, and thirteen palindromic SNPs (rs10127775, rs11917130, rs13270220, rs1982101, rs2273736, rs2934701, rs34038797, rs4812449, rs6535524, rs6547617, rs670179, rs7033052, rs850736), finally 488 SNPs were screened with F-values of 26.647-3373.567.

Based on the same process, the PCT group eliminated three incompatible alleles:rs11701383, rs150095649, rs7117331, twelve palindromic SNPs (rs10127775, rs10787518, rs10908505, rs10973700, rs11486499, rs13270220, rs1951856, rs34038797, rs484552, rs670179, rs7033052, rs725529), and finally 449 SNPs were included, with F-values 27.730-3004.535.

The MPV group excluded 8 incompatible alleles (rs113398097, rs113401351, rs139906758, rs34819411, rs35383388, rs371848983, rs70940197, rs71194512), 13 palindromic SNPs (rs1861435, rs216884, rs293365, rs35786616, rs4281782, rs496954, rs62194667, rs62376897, rs6547617, rs6690247, rs6763927, rs818586, rs850736), incorporating 481 SNPs with F-values 24.743-4037.326.

The PDW group excluded 3 (rs10984241, rs55950903, rs636918), 9 palindromic SNPs (rs11056314, rs2143139, rs3015483, rs34038797, rs342293, rs4678938, rs6086540, rs7002892, rs9888005) incorporated 383 individual SNPs with F-values 268.333-3507.926.

In this study, IVs for four platelet indices were selected according to established criteria. The F statistics for each selected IV exceeded 10, indicating a low risk of bias from weak IVs and thereby enhancing the analysis's reliability.

No SNPs related to the aforementioned confounding factors were found, ensuring the accuracy of the IV selection and the credibility of the analysis results.

Information on SNPs strongly associated with exposure in univariate MR analyses is presented in Supplementary Tables S2–S5.

Supplementary Table S6 presents a query table for IV confounding factors.

### 3.2 Two-sample MR analysis results

**Figure 2:**
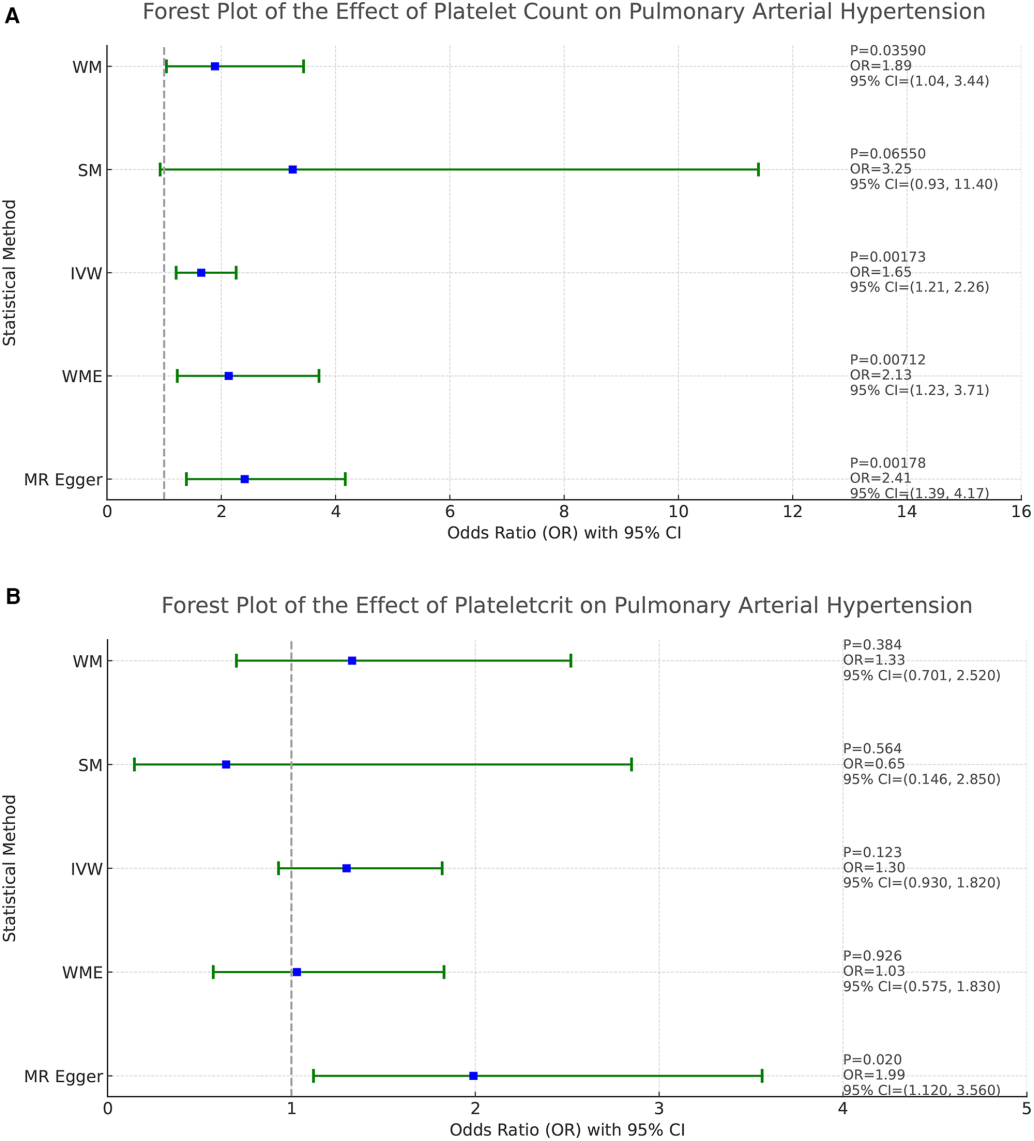
Forest plot of the result of two-sample Mendelian randomization. The causal effect was expressed as OR and 95% CIs of the estimates. (**A**) Forest plot of the effect of PLT on PAH. (**B**) Forest plot of the effect of PCT on PAH. (**C**) Forest plot of the effect of MPV on PAH. (**D**) Forest plot of the effect of PDW on PAH.

Based on the results of the analyses presented in Figure 2, we found a positive causal association only between PLT and PAH, whereas no clear causal link was shown between PCT, MPV, PDW and PAH.

**Figure:**
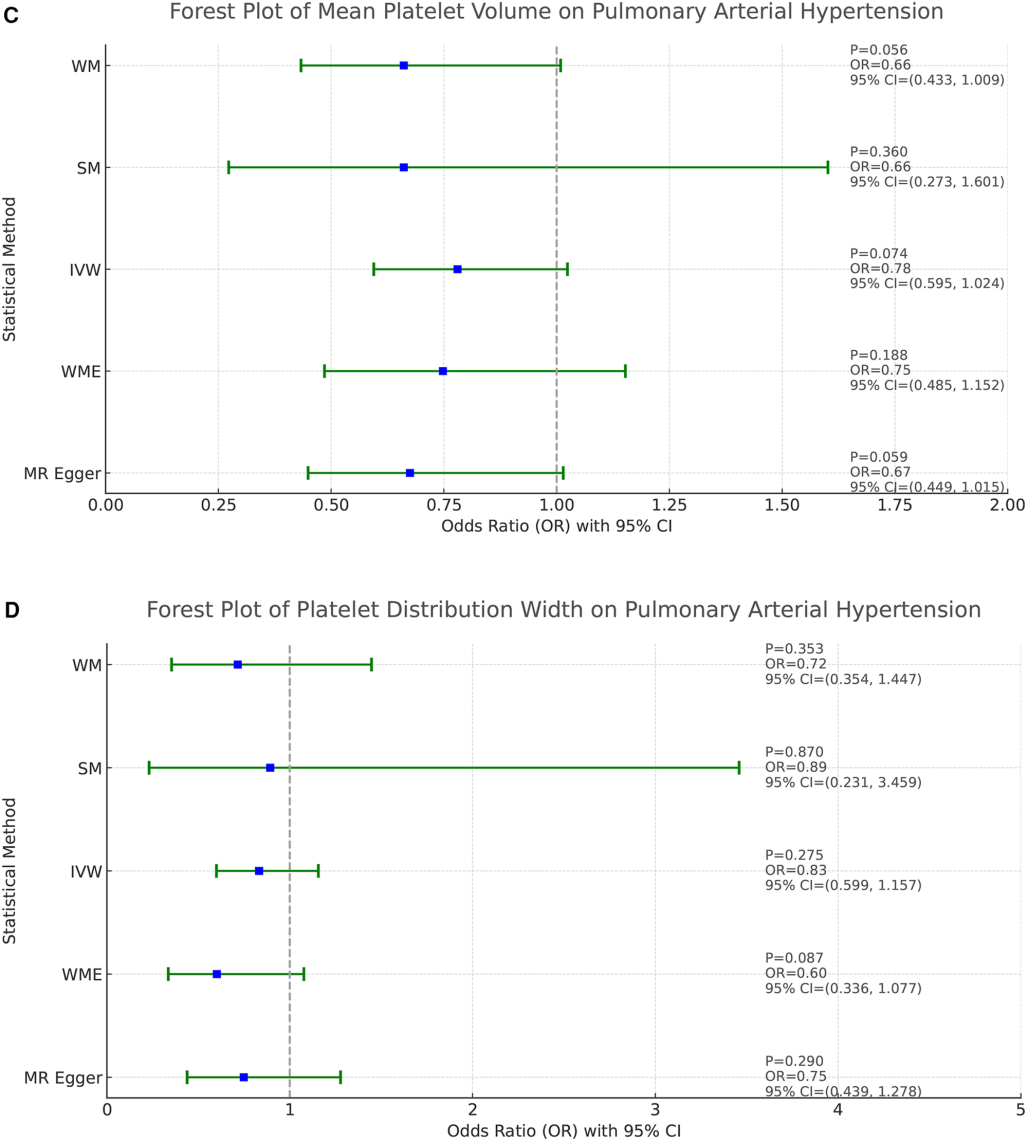


In the study addressing the relationship between PLT and PAH, five different statistical methods were used to analyze the results, which were broadly consistent, showing a positive correlation effect. After Bonferroni correction, the IVW, MR-Egger, and WME methods yielded statistical results at the significant level. Specifically, the statistical results were MR-Egger (OR = 2.410, 95%CI: 1.393–4.171, *P* = 0.0018), WME (OR = 2.134, 95%CI: 1.229–3.705, *P* = 0.0071), IVW (OR = 1.649, 95%CI: 1.206–2.256, *P* = 0.0017), SM (OR = 3.251, 95%CI: 0.930–11.38, *P* = 0.0655), and WM (OR = 1.895, 95%CI: 1.045–3.435 *P* = 0.0359). These results support that PLT may act as a risk factor for the development of PAH, and that increased platelet counts are associated with an increased risk of developing pulmonary hypertension. Refer to Figure 2A for a visual representation.

**Figure 3:**
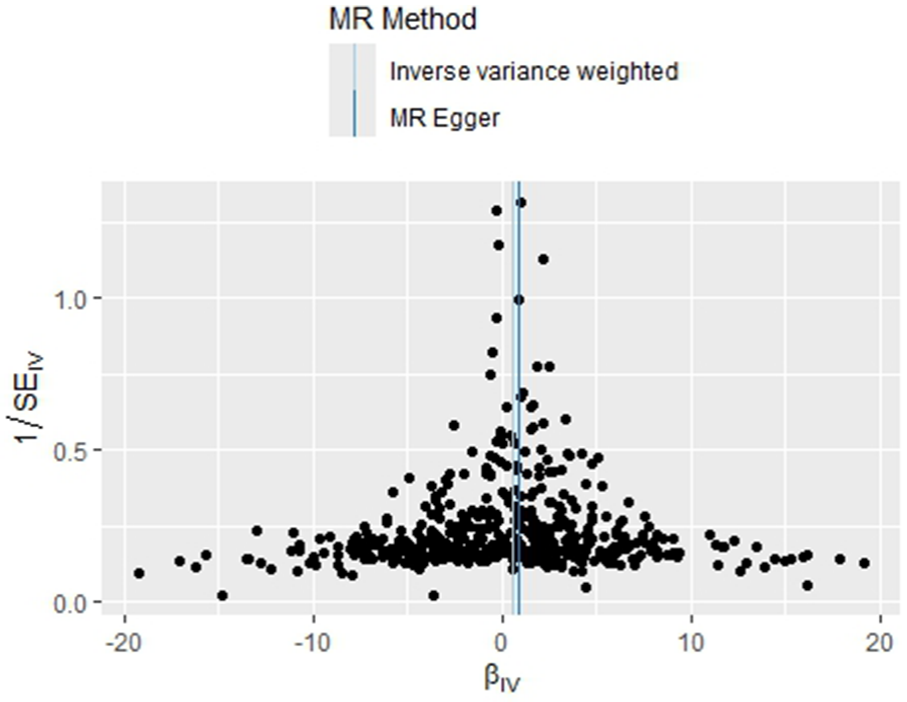
MR funnel plot for PLT on PAH.

The homogeneity of the results of the PLT and PAH analyses was further supported by the results of Cochran's *Q* test (*P* = 0.463). In addition, the global test of pleiotropy for MR-PRESSO also found no outlier SNPs (*P* = 0.568), indicating a lack of significant outlier heterogeneity in the MR analyses. The results of the intercept test for the MR-Egger regression showed no evidence of horizontal pleiotropy, with an intercept close to 0 (b_i = −0.0017, *P *= 0.879). The results of the above multiple analyses indicate that the selected IVs are unlikely to be affected by unobserved confounders. The results of the leave-one-out method showed that eliminating any of the SNPs did not significantly affect the estimation of the causal correlation, suggesting that the results of the MR analysis were robust. See Supplementary Figure S1. The distribution of causal effects in the funnel plot was largely symmetric, indicating no obvious bias (Figure 3).

### 3.3 MVMR analysis results

**Figure 4:**
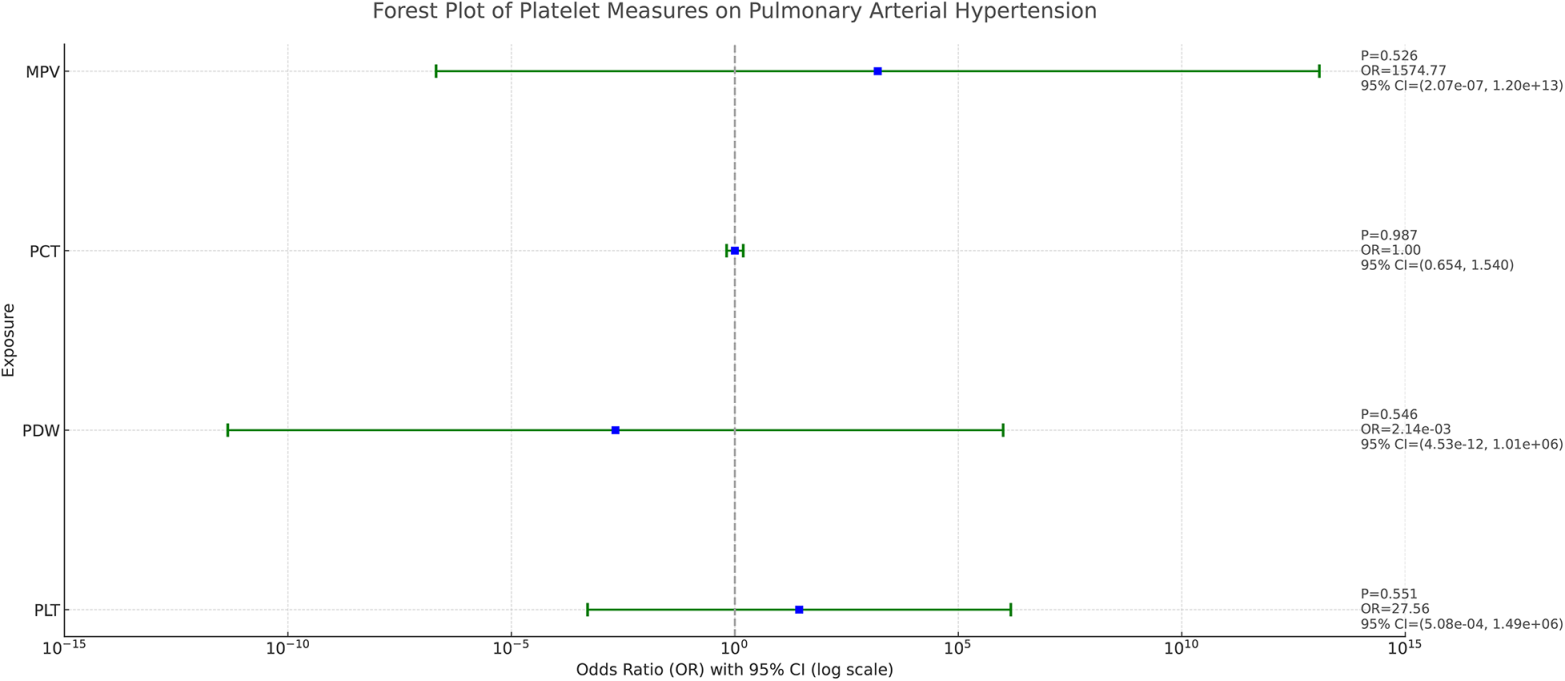
Forest plot of the causal effect of platelet indices on PAH in multivariable MR. The causal effect was expressed as OR and 95% CIs of the estimates.

As demonstrated in Figure 4, after adjusting the 4 platelet indices for each other, we found that none of them showed a clear causal relationship with PAH. This result suggests that the effects of these platelet indicators on PAH do not appear to be statistically independent after accounting for potential interactions between them.

**Figure 5:**
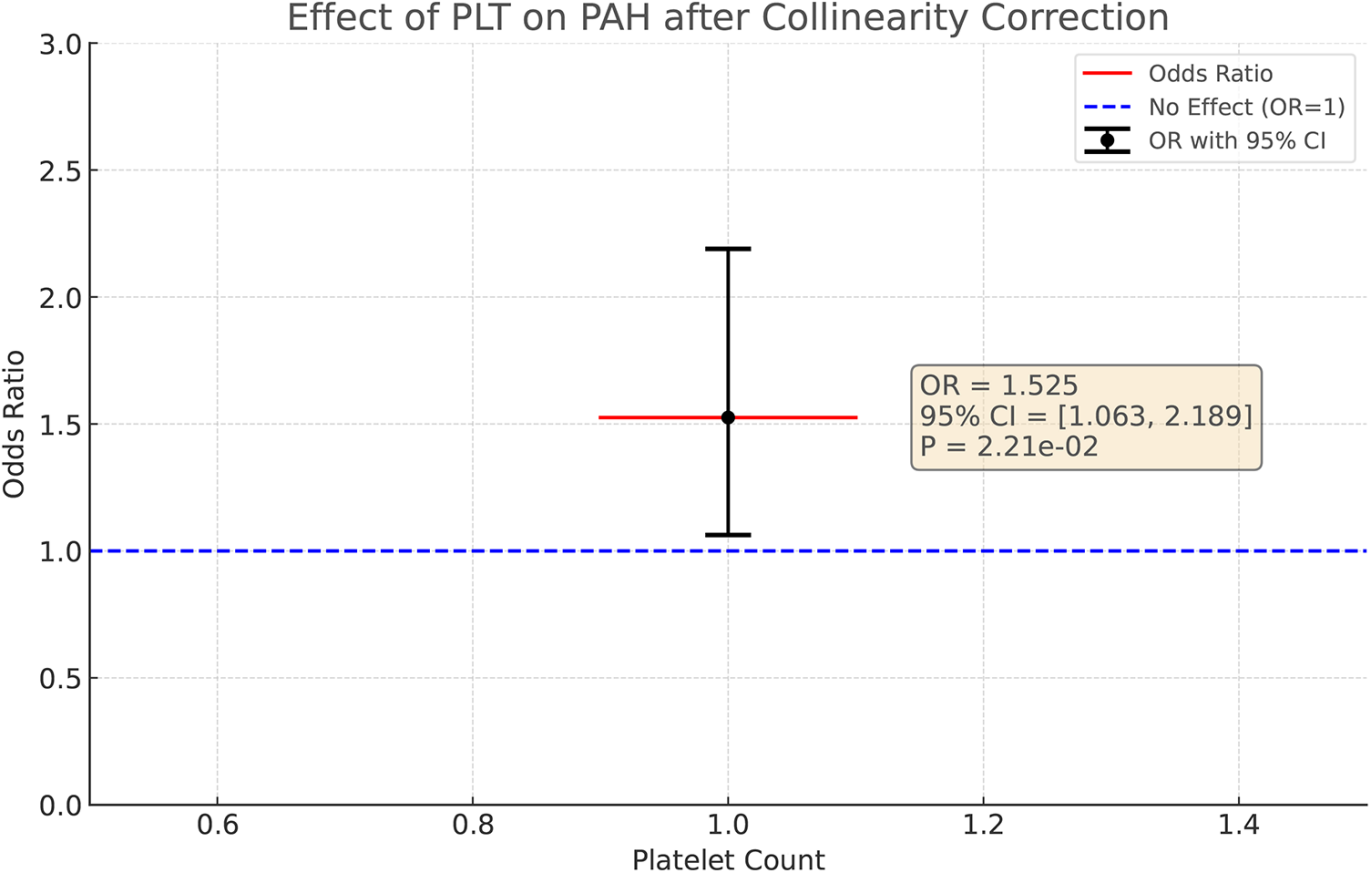
After colinear correction, the causal effect of PLT on PAH in multivariable MR. The causal effect was expressed as OR and 95% CIs of the estimates.

In view of the possible problem of covariance, we used the lasso regression test for covariance correction. With this method, we excluded the potential interference of PCT, PDW, and MPV on the results, thus allowing the independent effect of PLT on PAH to come to the fore, Figure 5. After correction, the effect of PLT on PAH revealed statistical significance (OR = 1.525, 95%CI: 1.063–2.189, *P* = 0.022), suggesting that, after excluding the PCT, MPV and PDW covariate effects, PLT may be an independent risk factor for the development of PAH.

Refer to Figure 5 for a visual representation.

## 4 Discussion

In this study, we investigated the potential causal relationship between four platelet indices (PLT, PCT, MPV, PDW) and PAH by Mendelian randomization. A significant causal relationship between PLT and PAH was revealed genetically using two-sample MR analysis. After correction for covariance by multivariate MR analysis, the effect of PLT on PAH remained significant, emphasizing that PLT may be an important independent risk factor for the development of PAH. When sensitivity analyses were performed, Cochran's *Q* test did not show significant heterogeneity in the selected IVs, and the MR-PRESSO test did not detect any outliers. In addition, neither the MR-Egger intercept test nor the MR-PRESSO test found evidence of horizontal pleiotropy, which further enhances the reliability and stability of our findings.

Our study revealed a significant causal relationship between PLT and PAH on a genetic level, showing a positive correlation with the risk of developing PAH. However, for PCT, PDW and MPV, no clear association with PAH was found at the genetic level. This finding does not completely align with some clinical observational studies.

A study conducted by Ya-Guo Zheng et al. included 82 patients with idiopathic pulmonary arterial hypertension (IPAH) and 82 age- and sex-matched controls to investigate the disease severity and prognosis of idiopathic IPAH, and demonstrated that MPV and PDW were significantly higher in IPAH patients than in age- and sex-matched controls, (11.4 ± 0.9 fl vs. 10.3 ± 0.9 fl and 14.3 ± 2.9% vs. 11. ± 9%; *P *< 0.001) (23). An observational study of hemostatic indices in patients with PAH showed that MPV was significantly higher in patients with PAH compared to healthy controls, but platelet counts were not significantly different (3). In a study evaluating prognostic indicators of combination therapy in patients with PAH, prognosis was analyzed from 22 variables by follow-up screening of 65 patients with IPAH, and only platelet levels were found to be associated with the risk of patient death (*P* < 0.01). Specifically, patients with lower pre-treatment platelet levels had a higher mortality rate than other patients (5). In addition to IPAH, PAH caused by other diseases also demonstrated some correlation with platelet parameters, a prospective study included 60 children with PAH combined with congenital heart disease and found that MPV and PCT were significantly higher in the PAH-CHD group compared to healthy controls and children with congenital heart disease without PAH, and these indices were strongly correlated with the severity of PAH (4). A study of PDW levels in patients with systemic lupus erythematosus-associated pulmonary arterial hypertension (SLE-PAH) vs. systemic lupus erythematosus simplex (SLE-non-PAH) showed that PDW levels were higher in the SLE-PAH group than in the SLE-non-PAH group (*P *= 0.023) (24).

These findings suggest that platelet indices may be closely associated with the pathogenesis of PAH, but the exact mechanisms remain incompletely understood. The increased number of activated platelets and the multiple cytokines they release may contribute to the pathogenesis of PAH (2). Research reports indicate a high frequency of thrombotic lesions in the histopathological classification of hypertensive pulmonary vascular disease (25). Thrombotic damage is common across various types of PAH (26), with thrombus formation closely linked to platelet activation, proliferation, and aggregation. In addition to causing thrombus formation, platelet activation triggers a series of changes in the vasculature of small pulmonary arteries, as well as in vascular endothelial cells. Specifically, activated platelets release a variety of growth factors, including PDGF and VEGF, which promote endothelial and smooth muscle cell proliferation (27). This mechanism is critical during vascular remodeling and thickening of the pulmonary vascular wall in pulmonary hypertension. *in vitro* experiments by Hai-Tao Yang et al. demonstrated that silencing the gene for EIF3A reduced PDGF-triggered proliferation, arrested the cell cycle, and down-regulated the expression of proliferation-associated proteins in pulmonary artery smooth muscle cells, ultimately reducing their proliferation and improving PAH (28).

Hypoxia commonly occurs in the pathogenesis of PAH. Chronic stimuli such as hypoxia, viral infections, and mechanical stress can induce an inflammatory response (29, 30). Inflammation is a key mechanism in pulmonary vascular remodeling and the pathogenesis of PAH. Platelets contain ATP, ADP, 5-HT, and growth factor- and cytokine-rich α-granules (31), which play a central role in inflammatory processes. These substances released by activated platelets exacerbate local inflammation and drive the proliferation of pulmonary artery smooth muscle cells, thereby aggravating PAH (32). It has been shown that platelets are more abundant in the lungs of hypoxic mice compared to normal mice, and that they are activated following hypoxia. Activated platelets aggregate and release vasoactive mediators, cytokines, and inflammatory factors, which exacerbate the progression of PAH. The increase in platelet counts and platelet-specific protein CD41 in lung sections from patients with advanced IPAH suggests that platelet proliferation and activation also play an important role in the progression of PAH in humans (33).

Therefore, in the development and progression of PAH, platelet activation can significantly induce changes in smooth muscle cells, endothelial cells, and extracellular matrix, as well as platelet aggregation, vasoconstriction, and remodeling of small pulmonary arteries, and other physiopathologic changes, which can alter pulmonary artery pressure levels. Increased pressure in the pulmonary arteries subsequently affects platelets, which is subsequently reflected in changes in the platelet index on clinical examination. Platelets play a key role in numerous pathophysiologic mechanisms of cardiovascular events. As indicators of platelet activation and functional status, different platelet parameters reveal changes in platelet number and function (34). These parameters are easy to measure and have been extensively utilized for the early diagnosis and prognosis of various cardiovascular and cerebrovascular diseases such as coronary heart disease (35, 36), hypertension (37, 38), heart failure (39), and stroke (40). According to existing observational studies, there is indeed an association between platelet markers and PAH, but the findings of observational studies are susceptible to confounding effects and reverse causality, and therefore, whether there is a causal relationship between changes in platelet markers and the development of PAH has not been conclusively established. MR studies have a unique advantage in exploring causal associations, and we used a well-established GWAS dataset to explore the relationship. In addition, we employed an MVMR approach to investigate the independent effects of the four platelet markers on the development of pulmonary hypertension, eliminating covariance effects to ensure result robustness. Based on our findings, the possible role of changes in platelet parameters in the development and progression of pulmonary hypertension should also be considered.

This paper also has several limitations. Firstly, our study only observed a causal relationship at the genetic level between PLT and the risk of PAH, while no causal relationship was found between PCT, MPV, PDW, and PAH, which is somewhat inconsistent with many published observational studies. Due to limitations in sample size, the number of SNPs identified with PAH as the exposure factor was too small, hence this paper did not conduct MR analysis on the effect of PAH on platelet indices nor could it verify reverse causality, Future research anticipates more comprehensive GWAS data and refined MR studies. And our GWAS datasets for platelet indices and PAH were derived from European ancestry, and results need to be confirmed in other ethnic groups. Lastly, as with all published MR studies, even with measures to identify and mitigate outlier variants, the possibility of unobserved pleiotropy affecting the results cannot be completely ruled out (41).

## 5 Conclusion

This MR study provides new evidence indicating that PLT is a major factor independently associated with PAH, while the relationships between PCT, MPV, PDW and PAH are not significant. These findings enhance our understanding of the role of platelets in the onset and progression of PAH, holding significant implications for clinical practice and the formulation of public health strategies.

Future studies should delve deeper into the specific pathophysiological mechanisms of platelets in the pathogenesis of PAH and look forward to more evidence based on stratified randomized controlled trials to assess the predictive value of metrics such as PLT in PAH and the efficacy of antiplatelet therapy in the PAH population.

## Data Availability

The datasets presented in this study can be found in online repositories. The names of the repository/repositories and accession number(s) can be found in the article/Supplementary Material.
