# Supplementary material for: Platelet indices and the risk of pulmonary arterial hypertension: a two-sample and multivariable Mendelian randomization study
Source: Front Cardiovasc Med. 2024 Aug 8;11:1395245. doi: 10.3389/fcvm.2024.1395245 (PMC11338760; doi:10.3389/fcvm.2024.1395245)
Supplement: Supplementary file 1 [file Table1.docx]

| Phenotype | Data source URL | GWAS ID | Sample ethnic origin | The number of SNPs | Number of cases | Sample size/ Number of controls | Data publication year |
| --- | --- | --- | --- | --- | --- | --- | --- |
| PLT | https://gwas.mrcieu.ac.uk/datasets/ebi-a-GCST90002402/ | ebi-a-GCST90002402 | European | 40,299,783 | NA | 408,112 | 2020 |
| PCT | https://gwas.mrcieu.ac.uk/datasets/ebi-a-GCST90002400/ | ebi-a-GCST90002400 | European | 40,299,196 | NA | 408,112 | 2020 |
| MPV | https://gwas.mrcieu.ac.uk/datasets/ebi-a-GCST90002395/ | ebi-a-GCST90002395 | European | 40,299,375 | NA | 408,112 | 2020 |
| PDW | https://gwas.mrcieu.ac.uk/datasets/ebi-a-GCST90002401/ | ebi-a-GCST90002401 | European | 40,300,122 | NA | 408,112 | 2020 |
| PAH | https://storage.googleapis.com/finngen-public-data-r10/summary_stats/finngen_R10_I9_HYPTENSPUL.gz | finngen_R10_I9_HYPTENSPUL | European | 19,814,121 | 248 | 289,117 | 2023 |
