# Supplementary figures and images for: Platelet indices and the risk of pulmonary arterial hypertension: a two-sample and multivariable Mendelian randomization study
Source: Front Cardiovasc Med. 2024 Aug 8;11:1395245. doi: 10.3389/fcvm.2024.1395245 (PMC11338760; doi:10.3389/fcvm.2024.1395245)

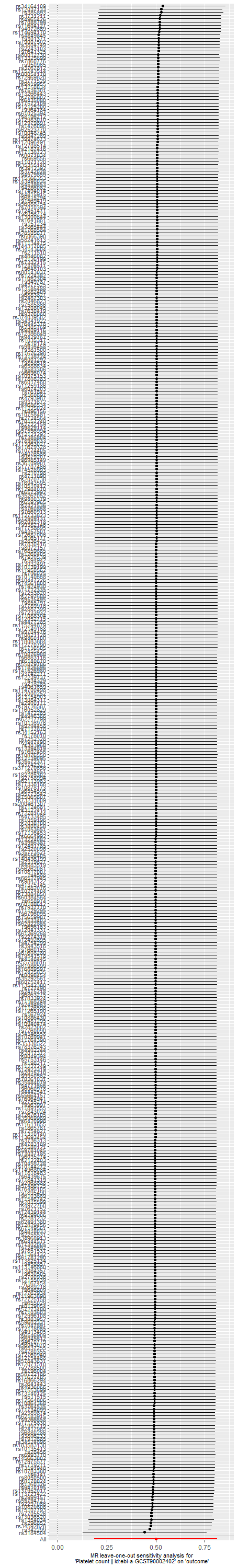

Supplement: Supplementary file 7 [file Image1.png]
